# An Unusual Case of Abdominal Pain

**DOI:** 10.1155/2012/827347

**Published:** 2012-09-30

**Authors:** Bobby Desai, Giuliano De Portu

**Affiliations:** ^1^Department of Emergency Medicine, University of College of Medicine, 1329 SW 16th Street, P.O. Box 100186, Gainesville, FL 32610-0186, USA; ^2^Department of Emergency Medicine, University of Florida College of Medicine, 1329 SW 16th Street, P.O. Box 100186, Gainesville, FL 32610-0186, USA

## Abstract

Renal calyceal rupture is a usual etiology of abdominal pain in the emergency department. We present a case of unexpected renal calyx rupture in a patient with symptomatology of renal colic. A discussion and review are provided.

## 1. Case

A 48-year-old female with history of fibromyalgia and hypertension was brought to our Emergency Department by Emergency Medical Services complaining of abdominal pain. She stated that she woke up with no symptoms and as the morning progressed she gradually started to develop left lower quadrant pain. She denied prior episodes of similar pain. The symptoms were localized to the LLQ and were nonradiating. She completed her menses 4 days ago. She denied any vomiting but did report nausea. The pain had been continuous in nature and on arrival was 10/10. She denied any vaginal bleeding or discharge. No fevers were reported. No urinary complaints were mentioned. Her last bowel movement was the morning of presentation and was reported as nonbloody. She denied diarrhea and reported that nothing improved her pain. She arrived in obvious discomfort.

On arrival, the patient's vital signs were temperature 35.4 degrees Celsius, heart rate 93 beats per minute, respiratory rate 32 breaths per minute, and blood pressure 134/82 mm Hg. Her review of system was positive only for abdominal pain. Physical exam was essentially normal including the genitourinary exam; pertinent positives included a tender abdomen in the left lower quadrant with involuntary guarding and no costovertebral angle tenderness.

Laboratory investigations included electrolytes and a complete blood count which were normal. The urinalysis was positive for protein only. The only laboratory test that was abnormal was lactate, which was elevated to 5.8 mmol/L.

Due to the inconclusive physical exam and the concerning lactate level, she was sent for a computed tomography scan for further evaluation of her abdominal pain. The findings were reported as a perinephric fluid collection consistent with calyx/forniceal rupture. A 3-4 mm nonobstructing stone was noted on the proximal ureter.

**Figure 1:**
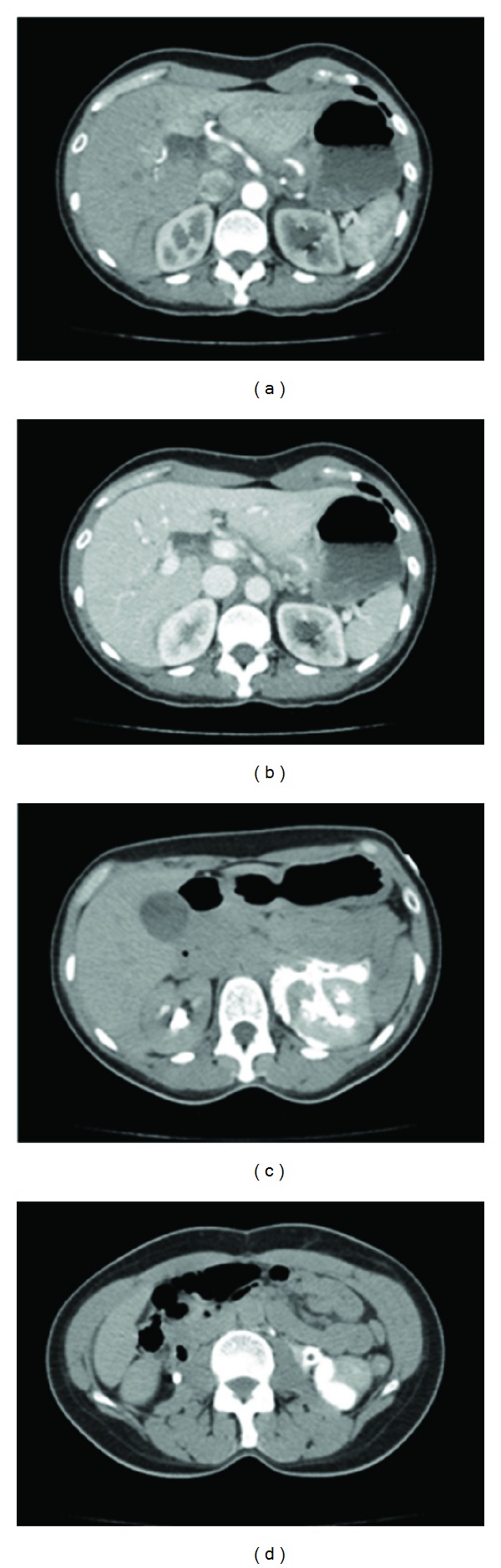
(a) CT arterial phase, (b) CT venous phase, (c) and (d) CT with delayed venous phase showing renal extravasation of IV contrast.

The urology service was consulted and recommended admission for observation, pain control, and placement of a ureteral stent and nephrostomy tube. The patient was subsequently discharged two days later without complication, see Figure 1.

## 2. Discussion

Spontaneous leakage of urine can occur without external trauma or urinary tract manipulation. The most common cause of this condition remains ureteral stones, but other causes have been reported, including prostatic enlargement, aortic aneurysm, and tumorous growths [1–4]. Obstruction of renal outflow tracts causes renal pelvic pressure to increase resulting in eventual rupture of the renal calyceal fornix. Direct blunt and penetrating trauma as well as renal instrumentation can also lead to rupture of the fornix. This is area of the kidney that is most susceptive to a rupture secondary to increase in pressure due to a stone [5].

Spontaneous rupture of a renal calyx may present with symptoms that are mild in nature, including mild abdominal pain or flank pain, as well as nausea and vomiting. However, on occasion, severe symptoms including hematuria and severe abdominal pain can develop. With continued leakage of urine, a urinoma—an encapsulated collection of urine—may be formed. Urine may also flow freely from the ruptured calyx and remain within the abdominal cavity, thus causing urinary ascites [6]. Complications of a ruptured renal calyx include infection of a urinoma or an infection around the kidney.

Diagnosis of a ruptured calyx can be made with ultrasound and computed tomography. Ultrasound can be used to quickly detect fluid around the kidney and the retroperitoneal areas; in addition, color duplex Doppler ultrasound can indirectly provide measurement of renal pelvis pressure [7, 8]. Computed tomography is also valuable in determining renal calyx rupture and can better delineate renal anatomy and presence of urinomas and can show perinephric stranding indicative of potential infection. Typically, delayed images are utilized in order for contrast to have time to leak out of the affected areas into retroperitoneal and perinephric spaces; images obtained 5–20 minutes after contrast administration are typically ideal [9].

Management of forniceal rupture involves several steps. For those ruptures that are caused by ureteral stones, the primarily goal is the relief of the acute obstruction by ureteral stenting and lithotripsy. Small leakages of urine can often be treated conservatively as they often spontaneously resolve. Larger urinomas or large leaks may require percutaneous drainage with a nephrostomy catheter and ureteral stent [10]. This diversion away from the leaking area of the kidney allows for healing of the renal collecting system [11].

In conclusion, spontaneous rupture of renal calyces is often not considered in the differential diagnosis of patients that present with abdominal pain and is often discovered during the routine evaluation of these patients with either ultrasound or computed tomography. Proper management of the condition is predicated upon its prompt recognition and urgent urologic consultation.
